# Chemical Composition and Antibacterial Activity of Essential Oils from *Ferula* L. Species against Methicillin-Resistant *Staphylococcus aureus*

**DOI:** 10.3390/molecules23071679

**Published:** 2018-07-10

**Authors:** Gulzhakhan A. Utegenova, Kyler B. Pallister, Svetlana V. Kushnarenko, Gulmira Özek, Temel Özek, Karime T. Abidkulova, Liliya N. Kirpotina, Igor A. Schepetkin, Mark T. Quinn, Jovanka M. Voyich

**Affiliations:** 1 Institute of Plant Biology and Biotechnology, Almaty 050040, Kazakhstan; gulzhakhan_utegen@mail.ru (G.A.U.); svetlana_bio@mail.ru (S.V.K.); 2 Faculty of Biology and Biotechnology, al-Farabi Kazakh National University, Almaty 050040, Kazakhstan; karime_58@mail.ru; 3 Department of Microbiology and Immunology, Montana State University, Bozeman, MT 59717, USA; ky.pallister@gmail.com (K.B.P.); liliya@montana.edu (L.N.K.); igor@montana.edu (I.A.S.); jovanka@montana.edu (J.M.V.); 4 Department of Pharmacognosy, Faculty of Pharmacy, Anadolu University, 26470 Eskisehir, Turkey; gulmiraozek@gmail.com (G.Ö.); tozek@anadolu.edu.tr (T.Ö.)

**Keywords:** *Ferula*, essential oil, antibacterial activity, methicillin-resistant *Staphylococcus aureus* (MRSA)

## Abstract

Essential oils (EOs) were obtained by hydrodistillation of various parts of *Ferula ovina* (Boiss.) Boiss., *Ferula iliensis* Krasn. ex. Korovin, and *Ferula akitschkensis* B. Fedtsch. ex Koso-Pol., collected in the flowering/budding and fruiting stages. Eight samples of EOs isolated from *F. ovina* and four samples from *F. akitsckensis* were analyzed by gas chromatography–mass spectrometry (GC-MS). The major constituents of *F. ovina* EOs were α-pinene (6.9–47.8%), β-pinene (1.5–7.1%), sabinene (0.1–20.5%), β-phellandrene (0–6.5%), *trans*-verbenol (0.9–7.4%), eremophilene (3.1–12%), and 6*Z*-2,5,5,10-tetramethyl-undeca-2,6,9-trien-8-one (0–13.7%). The major constituents of *F. akitsckensis* EOs were α-pinene (0–46.2%), β-pinene (0–47.9%), sabinene (0–28.3%), eremophilene (0–10.6), β-caryophyllene (0–7.5%), himachalen-7-ol (0–28.2%), and an himachalol derivative (0–8.3%). Samples of EOs from *F. ovina*, *F. iliensis*, and *F. akitsckensis* were evaluated for antibacterial activity against methicillin-resistant *Staphylococcus aureus* (MRSA) pulse-field gel electrophoresis type USA300 (LAC). EOs from *F. ovina* exhibited the highest antibacterial activity compared to samples from other *Ferula* spp., with the most potent EOs being isolated from roots at the flowering and fruiting stages and stems at the fruiting stage (IC_50_ values of 19.1, 20.9, and 22.9 µg/mL, respectively). Although EOs demonstrated concentration-dependent inhibition of MRSA growth, analysis of the major constituents (α-pinene, β-pinene, and sabinene) showed that they had low activity, suggesting that other components were likely responsible for the observed bioactivity of the unfractionated EOs. Indeed, correlation of the GC-MS data with antibacterial activity suggested that the putative components responsible for antibacterial activity were, either individually or in combination, eremophilene and *trans*-verbenol. Overall, these results suggest that the EOs from *F. ovina* could have potential for use as alternative remedies for the treatment of infectious diseases caused by MRSA.

## 1. Introduction

Methicillin-resistant *Staphylococcus aureus* (MRSA) is one of the main causative agents of skin and soft tissue infections. Infections caused by MRSA have limited treatment options since these strains are resistant to the entire class of β-lactam antibiotics. Vancomycin still remains the treatment of choice for serious MRSA infections [1]; however, vancomycin must be administered intravenously, which makes administration outside of hospital or clinical settings challenging. Additionally, *S. aureus* strains that have vancomycin intermediate resistance are prevalent and although rare, vancomycin resistant *S. aureus* strains have also been isolated [2]. Thus, there is an increased interest in finding alternative methods of treatment, including natural compounds such as essential oils (EOs), that are effective against bacterial infections [3,4]. The antimicrobial properties of EOs have been reported in several studies (reviewed in [5,6,7]), and combination of antibiotics with EOs targeting multidrug resistant bacteria could lead to new choices to overcome the problem of bacterial resistance [8,9]. Thus, EOs offer promise as an alternative treatment option. *Ferula* spp. are a good source of biologically active compounds, such as sesquiterpenes, terpenoid coumarins, and sulfur containing compounds [10,11,12,13,14,15,16]. The genus *Ferula* (Apiaceae) comprises ∼185 species distributed throughout Central Asia, the Mediterranean, and northern Africa, and many species of *Ferula* L. have been used in traditional medicine [13,17,18]. For example, one of the plant species in our study, *Ferula iliensis* Krasn. ex. Korovin, is a native plant of Kazakhstan that is widely used by the local population as an anti-inflammatory treatment [19]. The main constituents of most reported EOs from *Ferula* spp. exhibiting antimicrobial activity are monoterpenes, oxygenated monoterpenes, sesquiterpenes, and oxygenated sesquiterpenes [20,21,22,23]. Monoterpenes and sesquiterpenes are a frequently occurring group of compounds in EOs and have a broad spectrum of pharmacological properties, including antimicrobial activity [24,25,26,27,28]. Previously, we reported the chemical composition and immunomodulatory activity of EOs isolated from *Ferula iliensis* and *Ferula akitschkensis* [12,19]. Likewise, several sulfur compounds, including sec-butyl disulfide derivatives, were found in EOs and/or ole-gum resins obtained from various *Ferula* spp. [15,29,30,31,32]. The fruit oil of *Ferula latisecta* contains a high amount of polysulfide compounds, of which (*Z*)-1-propenyl sec-butyl disulfide (65.2%) and (*E*)-1-propenyl sec-butyl disulfide (6.8%) are the major constituents [33]. Monoterpene hydrocarbons dominated (85.7%) over all other compound groups in EOs from *F. akitschkensis* [12]. *Ferula ovina* (Boiss.) Boiss. is a fodder plant in Kazakhstan, and there is no information on the use of this plant in traditional medicine. However, *F. ovina* is a flavoring agent used as an ingredient in Iranian spices and condiments [34]. Aqueous extracts of *F. ovina* possess anti-spasmodic, anticholinergic, and smooth muscle relaxant activities [35], and antibacterial activity of *F. ovina* EOs against *S. aureus* was demonstrated by Syed et al. [36]. Radulovic et al. reported that bornyl 4-methoxybenzoate was one of the constituents of EOs from *F. ovina*, and it was shown that this compound induces hyperalgesia in mice [34].

In the present study, the chemical composition of EOs isolated from several samples of *F. ovina* and *F. akitschkensis* was evaluated. Antibacterial activity of EOs obtained from various parts of *F. ovina*, *F. iliensis*, and *F. akitschkensis* against MRSA was also assessed. Finally, three main constituents of the EOs (α-pinene, β-pinene, and sabinene) were evaluated for antibacterial activity.

## 2. Results and Discussion

### 2.1. Chemical Composition of Ferula EOs

EOs were isolated from various parts of *Ferula* species. The yield of EOs varied depending on the plant species and plant part. Specifically, the yields (*v*/*w*) of *F. ovina* EOs were: 0.97 (FOEO_I_), 0.16 (FOEO_Lfl_), 0.04 (FOEO_Sfl_), 0.95 (FOEO_Rfl_), 1.12 (FOEO_U/s_), 0.16 (FOEO_Lfr_), 0.03 (FOEO_Sfr_), and 0.78% (FOEO_Rfr_). The yields (*v*/*w*) of *F. akitschkensis* EOs were: 0.95 (FAEO_B_), 0.14 (FAEO_Lb_), 2.52 (FAEO_Rb_) and 2.24% (FAEO_Rfr_) (see abbreviations for the EOs in the footnote of Table 1). The chemical composition of two additional EOs isolated from umbels with seeds and stems at the fruiting stage of *F. akitschkensis* were reported previously [12]. Hydrodistillation of the umbels with seeds and stems produced 0.7 and 0.02% EOs, respectively [12]. The chemical composition of all EOs from *F. iliensis* were reported recently, and yields of their EOs varied from 0.4 to 1.1% [19].

The chemical composition of 8 EOs from *F. ovina* and 4 EOs from *F. akitschkensis* is summarized in Table 1, where the identified compounds are listed in order of their elution. In addition, the relative retention index (RRI_exp_) values obtained for the detected constituents are included for comparison with those values previously reported (RRI_lit_) for these compounds [37,38,39,40,41,42,43,44,45,46,47,48,49,50,51,52,53].

Analysis of the EOs from *F. ovina* revealed a total of 102 different constituents. FOEO_Lfl_ was found to be the most complex, with 62 constituents, while FOEO_U/s_, FOEO_Rfr_, FOEO_Lfr_, FOEO_Sfr_, FOEO_Sfl,_ FOEO_Rfl,_ and FOEO_I_ had 56, 53, 50, 45, 43, 43, and 41 constituents, respectively. Sabinene, α-pinene, β-pinene, eremophilene, β-phellandrene, *trans*-verbenol, and 6*Z*-2,5,5,10-tetramethyl-undeca-2,6,9-trien-8-one (all present at >5%) were the most common volatile constituents detected. Their concentrations varied depending on plant parts. For example, the highest content of α-pinene was identified in the inflorescence (35.1%), umbels with seeds (47.4%), and roots (47.8% and 46.5%). EOs isolated from the roots had a higher content of β-pinene and eremophylene compared to other parts of the plant. The content of sabinene was 20.5% in the inflorescence, whereas it was present only in trace amounts in the roots. GC analysis of the volatiles on a Lipodex G chiral column revealed the existence of enantiomeric pairs of α-pinene and β-pinene in FOEO_Sfr_, where we found (1*S*)-(−)-α-pinene (49%) and (1*S*)-(−)-β-pinene (29%).

It should be noted, that the main constituents previously reported for EOs from the seeds of *F. ovina* collected in China were polysulfide alkanes (86.3%), sesquiterpenoids (8.3%), and monoterpenoids (0.5%) [54]. EOs from leaves of *F. ovina* collected in Iran were mainly monoterpenes, specifically, α-pinene (50.0%) and limonene (11.5%) [55], which is similar to the chemical composition of EOs that we isolated from *F. ovina* (Table 1).

The volatile compounds identified in EOs isolated from buds, leaves, and roots at the budding stage and roots at the fruiting stage of *F. akitschkensis* are listed in Table 1. Analysis of these EOs revealed a total 105 different constituents. The most complex, FAEO_lb_, contained 51 constituents, while FAEO_Rb_, FAEO_Rfr_, and FAEO_B_ had 45, 45, and 37 constituents, respectively. Predominant constituents of the EOs obtained from buds and roots at the budding and fruiting stages and umbels with seeds were monoterpene hydrocarbons (70.6–95.2%), with the main compounds being α-pinene, β-pinene, and sabinene (Table 1), whereas EOs from stems at the fruiting stages were distinguished by a high percentage of myristicin (67.9%) and 2-himachalen-7-ol (7.9%) [12]. The existence of enantiomeric pairs in EOs isolated from umbels with seeds of *F. akitschkensis* was reported previously, where we found (1*S*)-(−)-α-pinene (95%), (1*S*)-(−)-β-pinene (94%), and (1*R*,5*R*)-(+)-sabinene (97%) [12]. A detailed chemical composition of EOs from *F. iliensis* was recently reported by our group, with the major constituents of the EOs from all parts of the plant being sulfur-containing compounds, including (*E*)-propenyl sec-butyl disulfide (15.7–39.4%) and (*Z*)-propenyl sec-butyl disulfide (23.4–45.0%) [19].

### 2.2. Antibacterial Activity of the EOs and Their Main Components

Eight samples of EOs isolated from *F. ovina*, seven samples from *F. iliensis*, and six samples from *F. akitsckensis* were evaluated for growth inhibitory activity in MRSA cultures, and the IC_50_ values are shown in Table 2. The results show that *F. ovina* EOs, especially FOEO_Rfl_, FOEO_Rfr_ and FOEO_Sfr_, had the highest growth inhibitory activity against MRSA, as compared to EOs from other *Ferula* spp. Low inhibitory activity was observed for all seven EOs isolated from *F. iliensis*. Likewise, EOs isolated from buds and leaves of *F. akitschkensis* had weak activity, while EOs isolated from other plant parts had no activity against the bacteria.

Based on chemical composition and biological activity of the EO samples tested (Table 1 and Table 2), three major constituents were selected for further analysis (α-pinene, β-pinene, and sabinene). The specific enantiomers were available from commercial sources: α-pinene and sabinene as racemic mixtures and the (−)-enantiomer of β-pinene. The effects of α/β-pinenes and sabinene on MRSA growth are presented in Table 2.

**Figure 1:**
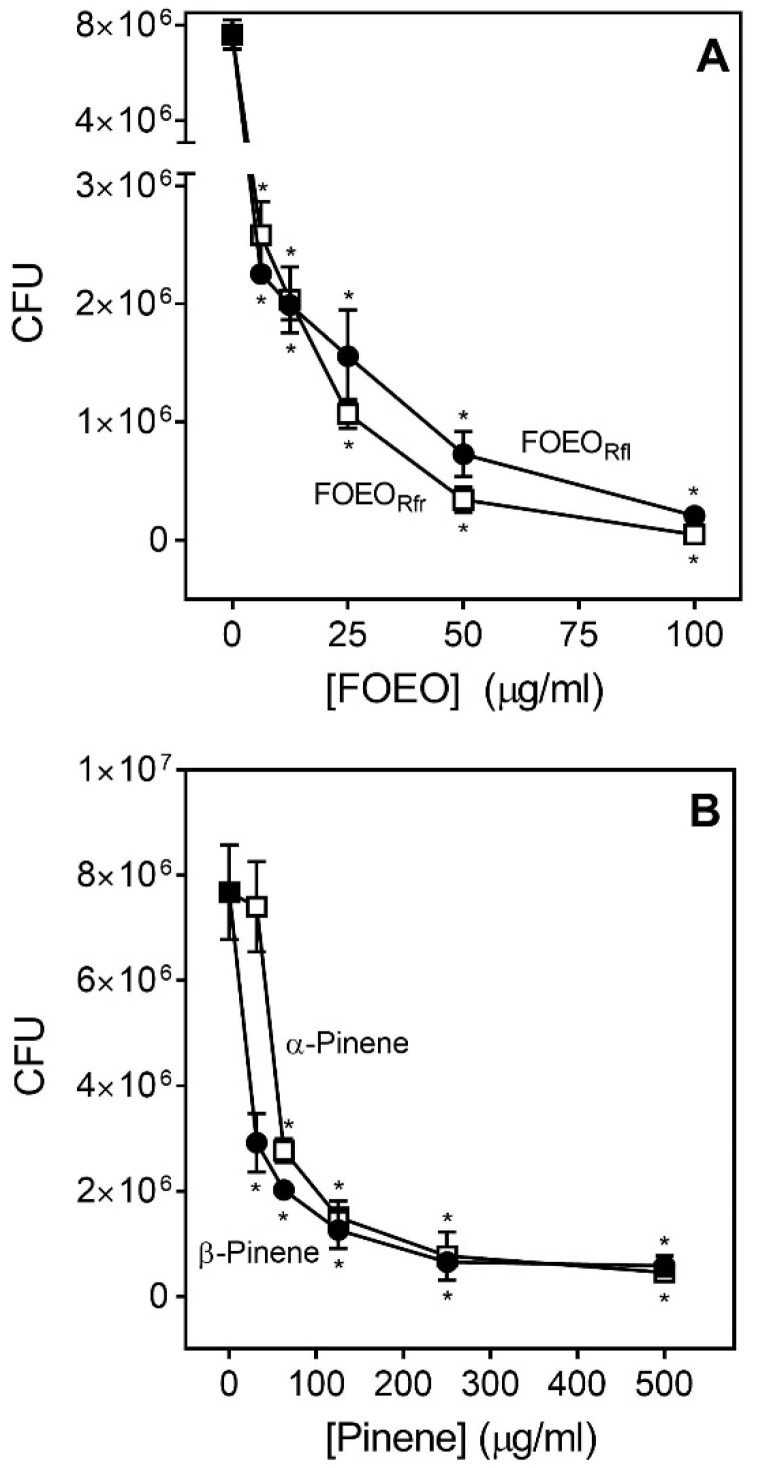
*Ferula ovina* essential oils (EOs) and their constituents inhibit MRSA growth in a dose-dependent manner. MRSA strain LAC USA300 was grown to mid exponential phase then resuspended in TSB (2 × 10^5^ CFU) and incubated with varied concentrations of EOs or constituents. CFUs were recovered following a 1 h incubation with the indicated concentrations of *F. ovina* EOs from roots at flowering (FOEO_Rfl_) and fruiting (FOEO_Rfr_) stages Panel (**A**) or EO constituents (±)-α-pinene and (−)-β-pinene Panel (**B**). * *p* < 0.001, as determined by one-way ANOVA with Dunnett’s test compared to LAC grown in DMSO. Data are from three separate experiments.

Antibacterial activity of the most active samples (FOEO_Rfl_, FOEO_Rfr_, and α/β-pinenes) against MRSA was also evaluated by enumerating the number of colony-forming units (CFU). Following a 1-h incubation of bacteria with the selected EOs, the bacteria were plated on solid media and incubated overnight. FOEO_Rfl_ and FOEO_Rfr_ significantly inhibited growth of MRSA, even at the lowest concentrations tested (6.25 µg/mL), and only a few bacterial colonies were observed at the highest tested concentrations (100 µg/mL) (Figure 1A). However, the individual constituents (±)-α-pinene and (−)-β-pinene demonstrated much weaker activity, even at the highest concentrations tested (Figure 1B).

To date, more than 70 species of *Ferula* have been chemically investigated [56,57,58]; however, there are only a few reports on the biological activity of EOs isolated from *Ferula* spp. In some studies, the bacteriostatic properties of EOs from *Ferula* spp. were associated with a high content of α-pinene and β-pinene or polysulfides [56]. EOs from *F. assa-foetida* contained sulfur compounds and had antimicrobial activity against *S. aureus*, *Staphylococcus epidermidis*, *Bacillus subtilis*, *Escherichia coli*, *Pseudomonas aeruginosa*, and *Klebsiella pneumoniae* [57], while EOs from *F. latisecta* were active against *S. aureus* and *Candida albicans* [33]. However, disulfides exhibited much lower antimicrobial activity than other sulfur containing compounds [58]. In the present studies, EOs from *F. iliensis*, which also mainly contain sulfur compounds, did not demonstrate a high level of antibacterial activity against MRSA. Likewise, Iranshahi et al. reported that EOs from the fruits of *F. latisecta*, which have a high content of polysulfides (mainly *sec*-butyl-(*Z*)-propenyl disulfide), exhibited only moderate antibacterial activity against *S. aureus* (ATCC 6538p) [33].

Although there are several reports on the antibacterial activity of EOs against *S. aureus* (e.g., see [59,60,61]), many of these studies involved high EO concentrations and only a few studies evaluated the effects of EOs at concentrations below 50 μg/mL. For example, Yamani et al. reported that EOs from *Ocimum tenuiflorum* at 2.25–2.5 μg/mL had bacteriostatic activity against two *S. aureus* strains, including MRSA [62]. The main volatile constituents of *O. tenuiflorum* EOs are monoterpenes and sesquiterpenes [62]. Likewise, EOs of *Aloysia polystachya* at 3.64, 7.28, and 29.13 μg/mL inhibited *S. aureus* ATCC 25923, *S. aureus* ATCC 29213, and MRSA, respectively [63]. The main compounds in *A. polystachya* EOs are carvone (78.9%) and limonene (14.2%) [63]. Here we found that EOs from *F. ovina* exhibited antibacterial activity against MRSA, with FOEO_Rfl_, FOEO_Rfr_, and FOEO_Sfr_ at concentrations of 19–22 μg/mL (Table 2). Thus, this is the first study showing effective antibacterial activity of EOs from *F. ovina* against a clinically-relevant MRSA strain (USA300).

Studies on the antimicrobial activity of monoterpenes showed that only the (+)-enantiomers of α-pinene and β-pinene had antibacterial activity against *C. albicans*, *Cryptococcus neoformans*, *Rhizopus oryzae*, and MRSA [27]. In our experiments, (±)-α-pinene and (−)-β-pinene demonstrated lower activity compared to unfractionated *F. ovina* EOs, and (±)-sabinene also had low activity. The highest percentage of the (+)-enantiomer of β-pinene was in FOEO_Sfr_. Although it could be suggested that this enantiomer was responsible for the antibacterial activity of unfractionated *F. ovina* EOs, some active EO samples (FOEO_Rfl_, FOEO_Rfr_, and FOEO_Sfr_) had lower levels of β-pinene (1.9 and 1.5%, respectively) (Table 1), which is not consistent with this conclusion. Additionally, α-pinene is present at high levels in *F. akitschkensis* EOs, yet these EOs had no antibacterial activity [12]. Thus, it is unlikely that (±)-α-pinene and (−)-β-pinene contribute significantly to the overall antibacterial activity observed.

The most active EOs from *F*. *ovina* were characterized by a high content of monoterpene hydrocarbons (9.1–71.1%), oxygenated monoterpenes (3.0–17.3%), and sesquiterpene hydrocarbons (7.9–25.9%). However, the various *F. akitsckensis* EOs, which had weak or no antibacterial activity, also had a similar range of monoterpene hydrocarbons (0.4% to 89.1%), oxygenated monoterpenes (1.4% to 3.6%), and sesquiterpene hydrocarbons (1.5% to 37.4%). In an effort to identify putative component compounds responsible for the observed antibacterial activity, we conducted a linear regression analysis based on antibacterial activity of the EOs evaluated and GC-MS data for their major (>5%) constituents (Table 1 and Table 2 and our previous publications [12,19]), as described previously [64]. Correlation was not analyzed for the amounts of 6*Z*-2,5,5,10-tetramethyl-undeca-2,6,9-trien-8-one, himachalol derivative, 10-epi-γ-eudesmol, (*E*/*Z*)-propenyl sec butyl disulfides, and myristicin because these compounds were found only in 1-7 samples of the EOs (see Table 1 and [12,19]). As a result of this analysis, relatively good correlations were obtained for *trans*-verbenol, eremophilene, α-pinene, the sum of α- and β-pinenes, and the total amount of monoterpene hydrocarbons and sesquiterpenes by plotting the logarithms of antibacterial activity (IC_50_) of the EOs versus their GC-MS data (Table 3).

**Figure 2:**
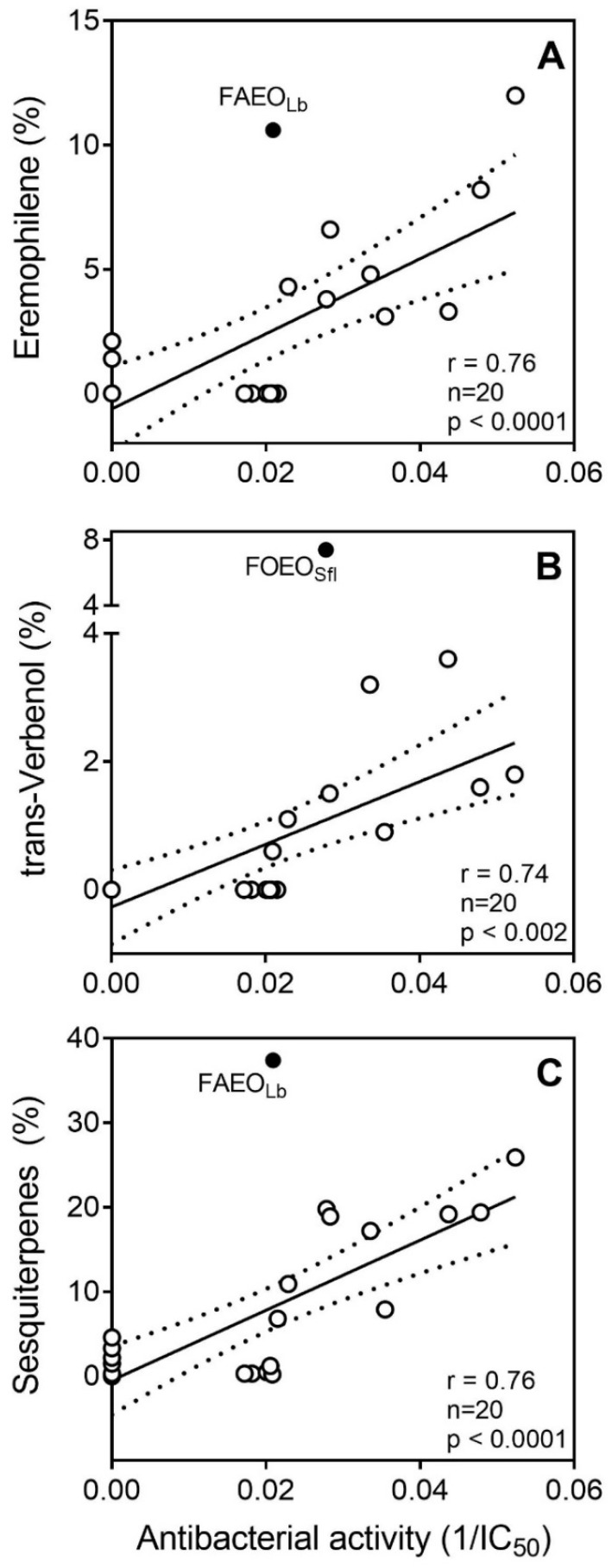
Plots of antibacterial activity versus the concentrations of eremophilene Panel (**A**), *trans*-verbenol Panel (**B**), and total sesquiterpene hydrocarbons Panel (**C**) in the EOs based on GC-MS data. Activities are represented as inverse (1/IC_50_) values to account for the four inactive EO samples from *F. akitschkensis*. These samples, indicated as closed circles, were omitted from the regression calculation and are shown as outliers. Dashed lines indicate area of the 95% confidence band. FAEO_Lb_, EO isolated from *F. akitsckensis* leaves at the budding stage; FOEO_Sfl_, EO isolated from *F. ovina* stems at the flowering stage.

To account for inactive EOs from *F. akitschkensis*, we also plotted the reciprocal values of antibacterial activity (1/IC_50_), where inactive samples were assigned a value of zero, and obtained a good linear correlation for *trans*-verbenol and eremophilene (Table 3 and Figure 2A,B). Antibacterial activity also correlated with the total quantity of sesquiterpenes present in the EO samples (Figure 2C), supporting the finding for eremophilene, an eremophilane-type sesquiterpene [65]. Moreover, various EOs isolated from *Verbenaceae* spp., which have a high amount of sesquiterpenes, were highly active against *S. aureus* (reviewed in [6]). Although we did not find a correlation with total amount of oxygenated monoterpenes (Table 3), our finding of *trans*-verbenol supports previous studies showing that oxygenated terpenoids may have more antimicrobial activity than some other EO constituents [66]. For the remaining major constituents, including α/β-pinenes and other chemical classes, no significant correlation between antibacterial activity and their concentrations in the EOs was found (Table 3). This is also consistent with previous studies showing that the presence of α/β-pinenes does not correlate with antimicrobial/antifungal activities [67,68]. Overall, *trans*-verbenol and eremophilene seem to represent reasonable targets for further analysis to define the anti-MRSA activity of the active EOs.

Unfortunately, these compounds are not commercially available and will require isolation, which is difficult due to their low concentrations, or possibly synthesis. Therefore, further studies are clearly warranted and are the focus of our ongoing research. 

In general, our analysis performed using two activity representations (LogIC_50_ and 1/IC_50_) suggests that anti-MRSA activity of the EOs could be attributed to the presence of eremophilene and/or *trans*-verbenol and/or their additive or synergistic effect with α/β-pinenes, sabinene, and other constituents. Thus, compounds present in the greatest proportions are not necessarily responsible for the largest share of the antibacterial activity, and involvement of less abundant constituents should be considered. For example, evaluation of the major compounds of *Piper hispidinervum* EOs showed that a low quantity of terpinolene increased the nematicidal effect of safrole when binary combinations of these compounds were tested [69]. However, the interactive effects of major active constituents of EOs from *Glossogyne tenuifolia* (linalool, 4-terpineol, α-terpineol, *ρ*-cymene) were additive instead of synergistic, as determined by checkerboard analysis with pathogenic bacteria, including *S. aureus* [70].

In conclusion, we report that EOs isolated from selected *Ferula* species have antibacterial activity against MRSA USA300, which is a relevant clinical strain. The most active EOs were isolated from *F*. *ovina* and were characterized by an abundance of monoterpene hydrocarbons, oxygenated monoterpenes, and sesquiterpenes. On the other hand, *F. iliensis* EOs had low antibacterial activity, suggesting that (*E*)-propenyl sec-butyl disulfide and (*Z*)-propenyl sec-butyl disulfide do not have significant activity against MRSA. Finally, *F. akitsckensis* EOs possessed weak or no antibacterial activity. Although EOs from *F. ovina* demonstrated concentration-dependent inhibition of MRSA growth, their major constituents (α-pinene, β-pinene, and sabinene) had low activity, suggesting that they were not responsible for the observed bioactivity of the unfractionated EOs. On the other hand, correlation of the GC-MS data with antibacterial activity suggested that the sesquiterpene hydrocarbon eremophilene and the oxygenated monoterpene *trans*-verbenol could be the constituents responsible for antibacterial activity. Further studies are clearly necessary to evaluate efficacy and elucidate the exact mechanisms by which EOs from *F. ovina* exhibit their antibacterial effects.

## 3. Materials and Methods

### 3.1. Chemicals and Materials

Three compounds found in EOs were obtained from commercial sources. (±)-α-Pinene was purchased from Santa Cruz Biotechnology (Dallas, TX, USA). (1S)-(−)-β-Pinene was from Alfa Aesar (Ward Hill, MA, USA). (±)-Sabinene was from Sigma-Aldrich Chemical Co. (St. Louis, MO, USA). The compounds were dissolved in dimethyl sulfoxide (DMSO) (Sigma-Aldrich Chemical Co.; 10 mM stock solutions) and stored at −20 °C. *S. aureus* was grown using tryptic soy broth (TSB) and tryptic soy agar (TSA) (EMD Millipore, Burlington, MA, USA) containing 0.5% glucose (Sigma-Aldrich).

### 3.2. Plant Material

*F. ovina* (Boiss.) Boiss., *F. iliensis*, and *F. akitschkensis* B. Fedtsch. ex Koso-Pol. were collected from the Almaty region of Kazakhstan in May–July 2015 at two stages: *F. ovina* and *F. iliensis* were collected during the flowering and fruiting stages, and *F. akitschkensis* was collected at the budding and fruiting stages. GPS coordinates: *F. iliensis* was collected at an altitude of 695 m above sea level (latitude, N 43°35′29′′; longitude, W 78°36′95′′). *F. ovina* was collected at an altitude of 1014 m above sea level (latitude, N 43°31′52′′; longitude, W 78°35′17′′). *F. akitschkensis* was collected at an altitude of 1525 m above sea level (latitude, N 43°16′70′′; longitude, W 77°42′86′′). Voucher specimens were deposited at the Institute of Plant Biology and Biotechnology (Almaty, Kazakhstan). Separately collected plant parts (buds, inflorescences, leaves, stems, roots, and umbels with seeds) were air-dried for 7–14 days at room temperature in shaded, well-aired rooms. Weighed samples were cut under laboratory conditions before hydrodistillation.

### 3.3. Isolation of EOs

EOs were obtained from air-dried plant material (30–60 g depending on plant parts) by hydrodistillation for 3 h using a Clevenger-type apparatus. For the hydrodistillation, the conditions accepted by the European Pharmacopoeia (European Directorate for the quality of Medicines, Council of Europe, Strasbourg, France, 2014) were applied. The yield of EOs was calculated on a dry weight basis. Solutions of the EOs were prepared in DMSO (10 mg/mL) for antibacterial evaluation and n-hexane (10% *w*/*v*) for gas chromatographic analysis.

### 3.4. Gas Chromatography–Mass Spectrometry (GC-MS) Analysis

Chemical composition of the EOs was determined as reported previously [11] using GC-FID and GC-MS. GC-MS analysis was performed with an Agilent 5975 GC-MSD system (Agilent Technologies, Santa Clara, CA, USA). An Innowax FSC column (60 m × 0.25 mm, 0.25 μm film thickness) was used with He as carrier gas (0.8 mL/min). GC oven temperature was kept at 60 °C for 10 min, increased to 220 °C at a rate of 4°C/min, kept constant at 220 °C for 10 min, and then increased to 240 °C at a rate of 1 °C/min. The split ratio was adjusted to 40:1, and the injector temperature was 250 °C. MS were collected at 70 eV with a mass range from m/z 35 to 450. GC analysis was performed using an Agilent 6890N GC system. To obtain the same elution order as with GC-MS, simultaneous injection was performed using the same column and appropriate operational conditions. Flame ionization detector (FID) temperature was 300 °C. The EO components were identified by co-injection with standards (whenever possible), which were purchased from commercial sources or isolated from natural sources. In addition, compound identities were confirmed by comparison of their mass spectra with those in the Wiley GC-MS Library (Wiley, New York, NY, USA), MassFinder software 4.0 (Dr. Hochmuth Scientific Consulting, Hamburg, Germany), Adams Library, and NIST Library. Confirmation was also achieved using the in-house “Başer Library of Essential Oil Constituents′′ database, obtained from chromatographic runs of pure compounds performed with the same equipment and conditions. A C8–C40 n-alkane standard solution (Fluka, Buchs, Switzerland) was used to spike the samples for the determination of relative retention indices (RRI). Relative percentage amounts of the separated compounds were calculated from FID chromatograms.

### 3.5. Chiral GC-MS Analysis

Chromatographic separation on a chiral column was performed for α-pinene, β-pinene, and sabinene. GC-MS analysis of the enantiomers in the oil was performed with an Agilent 7890 GC equipped with a FID and 5975 MSD with a triple-axis detector and an Agilent G 4513 autoinjector, integrated with a Gerstel CIS (Gerstel, Mülheim an der Ruhr, Germany; SEM Ltd., Istanbul, Turkey). Chiral separation was performed on a Lipodex G column (25 m × 0.25 mm × 0.125 μm film thickness; Macherey-Nagel, Düren, Germany) with He as the carrier gas (65 min at 5 mL/min, average velocity 77.985 cm/s). Injection quantity was 1 μL (10% in hexane). The temperature program for separation of α-pinene, β-pinene, and sabinene enantiomers was 50 min at 35 °C and then increased 40 °C/min to 200 °C for 10.875 min. Run time was 65 min. The split ratio was adjusted to 40:1, and the injector temperature was at 250 °C. FID temperature was 250 °C.

### 3.6. Bacterial Strain and Culture

MRSA pulse-field gel electrophoresis type USA300 cultures were grown in TSB containing 0.5% glucose. Overnight cultures of bacteria were diluted 1:200 in 20 mL TSB in a 125 mL flask and grown at 37 °C with shaking at 250 rpm. For all experiments, cultures were grown to mid-exponential growth phase (optical density at 600 nm [OD_600_] = 1.5).

### 3.7. Bacterial Growth Inhibition Assays

For analysis of antibacterial activity in culture, bacteria (2.5 × 10^7^ CFU/mL) were resuspended in TSB and incubated for 4 h at 37 °C with 5 different concentrations of EOs (6.25, 12.5, 25, 50, and 100 µg/mL) or with each of the constituents (α-pinene, β-pinene, and sabinene at 31.25, 62.5, 125, 250, and 500 µg/mL) in 96-well tissue culture plates. EOs or pure compounds diluted in DMSO were added to the wells (final concentration of DMSO was 1%). DMSO was used as a negative control. The growth suppression of bacteria was monitored as absorbance (λ = 600 nm) every 5 min for 4 h using a SpectraMax 190 microplate reader. Spectinomycin was used as positive control, and 50 µg/mL of this antibiotic completely inhibited bacteria growth.

For analysis of EO or constituent effects on bacterial survival, bacteria (2 × 10^5^) were resuspended in TSB and added to 96-well tissue culture plates with different concentrations of compounds diluted in TSB. The plates were incubated for 1 h at 37 °C, and the samples were plated onto TSA in Petri dishes. At the indicated time points, samples were serially diluted (1:10) in water, and CFU were enumerated the next day, as reported previously [71]. 

### 3.8. Statistical Analyses

The inhibitory effect of EOs against MRSA USA300 (LAC) was determined by calculation of the inhibitory concentration values (IC_50_) as the mean ± S.D. of three independent experiments. To calculate median IC_50_, curve fitting was performed by nonlinear regression analysis of the dose–response curves generated using Prism 7 (GraphPad Software, Inc., San Diego, CA, USA). One-way analysis of variance (ANOVA) was performed on the datasets, followed by Dunnett’s test. For correlation analyses, the Spearman rank correlation coefficient (*r*) was calculated.

## Figures and Tables

**Table 1:** Composition of the volatile compounds identified in the essential oils from different parts of *F. ovina* and *F. akitschkensis*.

RRI_exp_	RRI_lit_	Compound	Concentration in EOs (%) ^a^											
			FOEO_I_	FOEO_Lfl_	FOEO_Sfl_	FOEO_Rfl_	FOEO_U/s_	FOEO_Lfr_	FOEO_Sfr_	FOEO_Rfr_	FAEO_B_	FAEO_Lb_	FAEO_Rb_	FAEO_Rfr_
1032	1032	α-Pinene ^§^	35.1	10.3	15.0	47.4	47.8	6.9	7.6	46.5	25.0		36.4	46.2
1035	1035	α-Thujene ^§^	1.2	0.3	tr			0.6	tr		0.9			
1072	1070	α-Fenchene ^§^											tr	
1076	1076	Сamphene ^§^	0.2	0.6	tr	0.8	0.4	0.5	tr	0.5	0.2		0.5	1.0
1118	1118	β-Pinene ^§^	6.0	2.6	3.6	1.9	7.1	1.7	1.5	6.7	11.1		47.9	28.6
1132	1132	Sabinene ^§^	20.5	5.5	2.0	tr	6.5	3.2	tr	tr	28.3		tr	tr
1158	1137	Thuja-2,4(10)-diene ^§^			tr	0.3	tr	0.1		0.2			tr	0.1
1159	1159	δ-3-Carene ^§^		0.2							0.2		3.9	tr
1174	1175	Myrcene ^§^	0.8	0.2		0.4	1.0	0.4	tr	0.5	0.6		3.1	8.3
1176	1176	α-Phellandrene ^§^									tr		0.1	
1188	1188	α-Terpinene ^§^	0.3				0.2			tr	0.2		tr	0.1
1203	1204	Limonene ^§^	0.6	0.7	tr	0.3	0.6		tr	0.3	0.4		1.8	2.2
1218	1218	β-Phellandrene ^§^	3.9	1.7	tr		6.5	0.6	tr		0.4		0.8	2.2
1246	1246	(*Z*)-β-Ocimene ^§^	0.1		tr		tr	1.6	tr	tr			tr	
1255	1255	γ-Terpinene ^§^	0.7	0.2	tr		0.3	0.2		tr	0.6		tr	0.1
1266	1266	(*E*)-β-Ocimene ^§^			tr		tr	0.3						
1280	1280	*p*-Cymene ^§^	0.8	1.3	1.1	0.2	0.6	0.7	tr	0.4	2.5	0.4	0.5	0.2
1286		Isoterpinolene											0.1	
1290	1290	Terpinolene ^§^	0.3				0.1	0.1	tr	tr	0.2		0.1	0.1
1439	1477	γ-Campholene aldehyde ^§^						0.1	tr	tr				
1474		*trans*-Sabinene hydrate ^§^	0.4	0.2			0.2	0.1			0.6	0.4		
1482	1482	Longipinene ^§^	0.2	0.4	tr	0.8	0.3	0.4	tr	0.5	0.2	0.5		
1482	1464	Fenchyl acetate ^§^											0.1	0.2
1487	1487	Citronellal ^§^		0.4										
1492	1485	Cyclosativene ^§^												0.1
1493	1493	α-Ylangene ^§^	0.3	1.0	0.8	1.3	0.7	1.2	1.6	0.6	0.5	0.9	tr	
1497	1497	α-Copaene ^§^										0.2	0.3	0.3
1499	1500	α-Campholene aldehyde ^§^		0.7	1.6	0.5	tr	0.4		0.3				
1506	1506	Decanal ^§^		0.7				0.4						
1512	1497	Longicyclene ^§^				tr	tr			0.1		0.2		
1525	1528	Cyperene ^§^												0.1
1544	1545	α-Gurjunene ^§^											0.1	tr
1549	1549	β-Cubebene ^§^											0.1	0.1
1553	1553	Linalool ^§^		0.3				0.5						
1556	1571	*cis*-Sabinene hydrate	0.3				0.2				0.4	0.6		
1571	1573	*trans*-p-Menth-2-en-1-ol ^§^	0.1	0.2			0.1				0.2	0.4		
1586	1586	Pinocarvone ^§^		0.4	0.8		0.1	0.2	tr	0.1	0.2			
1587		1,7-Diepi-β-cedrene				0.2								
1589	1565	Aristolene		0.2		0.4			tr					0.1
1591	1592	Bornyl acetate ^§^		0.1									0.2	0.6
1595	1588	Isothymol methylether								0.2				
1596	1590	*trans*-β-Bergamotene				0.2				0.1	tr			
1596	1596	α-Guaiene ^§^											tr	0.1
1600	1600	β-Elemene ^§^				tr		0.1	tr					
1604	1598	Thymol methyl ether		0.5	0.9	tr		0.3	tr	1.3			0.1	0.2
1610	1611	Calarene				0.5								tr
1611	1611	Terpinen-4-ol ^§^	1.8	1.4	0.7		1.2	1.1			1.8	0.2		tr
1612	1612	β-Caryophyllene ^§^	0.1	0.1	tr	2.6		2.9	tr	1.3	1.8	7.5		
1628	1629	Aromadendrene ^§^											tr	0.1
1638	1638	*cis*-p-Menth-2-en-1-ol									0.1	0.2		
1648	1648	Myrtenal ^§^			1.7	0.2				0.2			0.1	
1650	1650	γ-Elemene ^§^	0.1	0.6			0.6		tr	tr				
1659	1668	γ-Gurjunene ^§^											0.1	
1661	1663	α-Himachalene ^§^	0.6	0.8	1.1	2.0	0.3	0.7	1.2	1.6	1.2	3.2	0.1	
1661	1661	*trans*-Pinocarvyl acetate ^§^										0.2	0.2	0.2
1663	1663	*cis*-Verbenol ^§^		1.3	1.1	tr	tr	0.4		0.5				
1668	1668	(*Z*)-β-Farnesene ^§^											0.2	0.1
1672	1671	*trans*-Pinocarveol ^§^		tr	1.8	0.6	tr	0.3		0.1	0.1			
1687	1689	α-Humulene ^§^				0.2	tr	0.6		0.3		0.6		
1683	1683	*trans*-Verbenol ^§^	0.9	3.2	7.4	1.8	1.1	1.5	3.6	1.6		0.6		
1697	1718	4,6-Guaiadiene										0.6		
1704	1704	γ-Muurolene ^§^							tr	0.1		0.4		
1704	1704	Myrtenyl acetate											0.2	0.2
1706	1706	α-Terpineol ^§^									0.2		0.1	0.1
1711	1708	γ-Himachalene	0.7	0.8	1.1	1.7	0.7	0.8	1.3	1.5	1.3	3.9		
1722	1722	Dodecanal ^§^		0.6										
1725	1725	Verbenone			tr	0.4								
1726	1726	Germacrene D ^§^											0.1	0.1
1730		Cadina-3,5-diene												0.5
1739	1740	β-Himachalene	0.8	0.9	1.6	2.3	0.9	0.9	1.6	2.0	1.5	4.6		
1740	1740	Valencene ^§^					0.1							
1740	1740	α-Muurolene ^§^												0.2
1741	1741	β-Bisabolene		0.8		tr							0.1	tr
1742	1743	β-Selinene ^§^							3.3					
1743	1743	Eremophilene ^§^	3.1	4.8	3.8	12.0	4.3	6.6	3.3	8.2		10.6		
1744	1740	α-Selinene ^§^												0.1
1750		Dauca-8,11-diene												0.1
1754		Himachala-2,4-diene*										0.6		
1768	1761	*cis*-α-Bisabolene ^§^										0.7		
1771	1773	γ-Bisabolene ^§^				0.7				0.1				
1771	1771	*cis*-Piperitol ^§^										0.2		
1772	1774	Citronellol ^§^		1.5			tr		1.8					
1773	1774	δ-Cadinene ^§^				1.0	tr			0.1		0.2	0.2	
1783	1783	β-Sesquiphellandrene ^§^												0.1
1784	1786	(*E*)-α-Bisabolene								0.1		0.7		
1786	1786	*ar*-Curcumene ^§^									0.2		tr	tr
1788	1782	1-Decanol ^§^		0.2										
1796	1790	Selina-3,7(11)-diene					tr			0.2			0.1	
1804	1804	Myrtenol ^§^	0.1	0.5	1.3		0.1		tr	0.2			0.1	0.1
1849	1849	Cuparene ^§^											0.1	0.1
1853	1853	*cis*-Calamenene										0.5	tr	
1854	1853	Germacrene B ^§^	1.0	1.9	0.9		1.9	4.7	2.2	0.3	0.1	1.0		
1864	1864	*p*-Cymen-8-ol ^§^									tr	0.4		
1868	1868	*(E)*-Geranyl acetone ^§^											0.3	0.2
1869		Neophytadiene										0.6		
1871	1878	Neryl isovalerate	0.1											
1878	1878	2,5-Dimethoxy-*p*-cymene							tr	0.1				
1882		α-Dehydro-ar-himachalene		0.3	0.6		tr		tr	0.1				
1888	1888	ar-Himachalene ^§^	1.0	2.7	4.0		0.9		4.7	1.7				
1925		γ-Dehydro-ar-himachalene			1.3		0.1	tr		0.1				
1933	1930	Neryl valerate	0.2	1.9	tr		tr	1.8						
1941	1941	α-Calacorene-I		0.2			0.1			0.2		0.2		
1956	1954	(*E*)-β-Ionone										0.2		
1973	1973	1-Dodecanol ^§^		0.2										
1984	1984	α-Calacorene-II				1.2	tr			0.1				
2001	2001	Isocaryophyllene oxide				tr		0.5		0.3				
2004		Oxidohimachalene		0.2	tr	tr	tr	0.3	tr					
2008	2008	Caryophyllene oxide ^§^	0.1	1.4	1.4	0.5	0.1	2.6	1.5			0.7		
2030	2029	Methyl eugenol ^§^	0.4	0.2				0.3						0.1
2044		6,7-Epoxy-himachalene	0.1	0.6	1.1	0.2	0.3	0.3	1.4	0.3		0.2		
2068		α-Copaene-8-ol *		1.9										
2071	2071	Humulene epoxide II						0.3		0.1				
2080	2033	Junenol					0.1							
2131		1-α-(*H*)-himachal-4-en-1-β-ol				0.3	0.1	0.3		0.2	0.1			
2165	2131	Hexahydro-farnesylacetone ^§^										0.3		
2169		DMPF										0.4		
2179	2100	6-epi-Cubenol		0.7		0.4	0.2	0.5		0.4	0.2			
2219	2214	Torreyol		0.5		0.2		0.2						
2219		Dimyrcene II-a												0.1
2232	2232	α-Bisabolol	0.6	1.3		0.6	0.4		3.1	0.6				
2240	2256	epi-α-Bisabolol			1.5			2.1						
2245	2245	Elemicine ^§^											0.1	0.2
2248	2246	Himachalol ^§^						2.4						
2249		β-Himachalol *										1.5		
2252		Himachalol derivative *										8.3		
2254		2-Himachalen-7-ol	2.4	2.9	5.4	2.1	2.1		5.8	2.3	6.9	28.2		
2256	2256	Cadalene		0.2	2.2					tr		0.3	tr	
2296	2296	Myristicine ^§^											0.9	4.1
2273	2273	Allohimachalol ^§^	0.1				0.1		3.2	0.3	0.3	1.0		
2278	2278	Torilenol	0.7	2.5	3.0		1.0			1.0				
2280		(*E*)-Longipinane *		1.5										
2300	2300	Tricosane ^§^										tr		
2303		8,9-Dehydroneoisolongifolene *			2.4									
2304		TMCMP		1.7										
2308	2332	Khusinol	1.0					2.2						
2376		10-Hydroxy-calamenene	0.2				0.2		1.9					
2456		Oxygenated sesquiterpene *							8.4					
2467		GTO *							3.7					
2468		Marsupellol				3.1								
2482		Dauca-8(14),11-dien-9 α-ol		2.0				2.0						
2500	2500	Pentacosane ^§^							1.2			0.4		
2533	2533	γ-Costol										0.6		
2542		Eudesma-4(15),7-dien-1-ol		0.5								0.6		
2565		1-Hexadecanol										0.4		
2575		10-Hydroxy-calamenene isomer *										0.5		
2606	2607	β-Costol										0.4		
2620	2619	Phytol ^§^		0.6				0.6				2.7		
2700	2700	Heptacosane ^§^										1.0		
2900	2900	Nonacosane ^§^										4.4		
2931	2931	Hexadecanoic acid ^§^	1.3	1.0			0.6	1.7	tr	0.9	0.5	0.5		
2931		TMUTO *		4.7	4.7				13.7					
**Total % Based on Chemical Class**			**89.2**	**75.8**	**75.9**	**92.4**	**90.1**	**59.2**	**79.5**	**85.5**	**89.0**	**93.9**	**99.1**	**97.6**
Monoterpene hydrocarbons			70.5	23.6	21.7	51.3	71.1	16.9	9.1	55.1	70.6	0.4	95.2	89.1
Oxygenated monoterpenes			3.9	12.6	17.3	3.5	3.0	6.7	5.4	4.6	3.6	3.2	1.4	1.8
Sesquiterpene hydrocarbons			7.9	17.2	19.8	25.9	10.9	18.9	19.2	19.4	6.8	37.4	1.5	2.2
Oxygenated sesquiterpenes			5.2	13.1	12.4	7.4	4.6	13.7	30.9	5.5	7.5	42.3	0.0	0.0
Miscellaneous compounds			1.7	9.9	4.7	4.3	0.6	3.0	14.9	0.9	0.5	10.5	1.0	4.5

^a^ The data are presented as a relative percentage by weight for each component in EOs isolated from *F. ovina* inflorescences (FOEO_I_), leaves at the flowering stage (FOEO_Lfl_), stems at the flowering stage (FOEO_Sfl_), roots at the flowering stage (FOEO_Rfl_), umbels with seeds (FOEO_U/s_), leaves at the fruiting stage (FOEO_Lfr_), stems at the fruiting stage (FOEO_Sfr_), and roots at the fruiting stage (FOEO_Rfr_) and EOs isolated from *F. akitsckensis* buds (FAEO_B_), leaves at the budding stage (FAEO_Lb_), roots at the budding stage (FAEO_Rb_), and roots at the fruiting stage (FAEO_Rfr_). RRI_exp_, relative retention indices calculated against n-alkanes, % calculated from FID data. RRI_lit_, published RRI values for the volatile compounds [37,38,39,40,41,42,43,44,45,46,47,48,49,50,51,52,53]. ^§^ Compounds identified by co-injection. Trace amount (tr) were present at <0.1%. * Tentatively identified from the Wiley mass spectrum library. DMPF, 3,4-dimethyl-5-pentylidene-2(5*H*)-furanone; TMCMP (*1E*)-1-[2,6,6-trimethylcyclohex-1-enyl]-3-methyl-1,4-pentadien-3-ol; GTO, germacra-4(15),5,10(14)-trien-1a-ol; TMUTO, 6*Z*-2,5,5,10-tetramethyl-undeca-2,6,9-trien-8-one.

**Table 2:** Antibacterial screening of the EOs from *F. ovina*, *F. iliensis*, and *F. akitschkensis* and their major constituents against MRSA strain LAC.

Plant Species	Part of Plant	EO Name	IC_50_ (μg/mL)
*F. ovina*, flowering stage	inflorescence	FOEO_I_	28.2 ± 2.8
	leaf	FOEO_Lfl_	29.8 ± 2.9
	stem	FOEO_Sfl_	35.9 ± 2.0
	root	FOEO_Rfl_	19.1 ± 2.9
*F. ovina*, fruiting stage	umbels with seeds	FOEO_U/s_	43.7 ± 4.1
	leaf	FOEO_Lfr_	35.3 ± 1.9
	stem	FOEO_Sfr_	22.9 ± 0.8
	root	FOEO_Rfr_	20.9 ± 1.2
*F*. *iliensis*, flowering stage	inflorescence	FEO_Fl_	55.0 ± 10.2
	leaf	FEO_Lfl_	94.3 ± 11.1
	stem	FEO_Sfl_	79.1 ± 8.9
	root	FEO_Rfl_	58.1 ± 6.1
*F. iliensis*, fruiting stage	umbels with seeds	FEO_Fr_	49.8 ± 3.8
	stem	FEO_Sfr_	48.0 ± 2.0
	root	FEO_Rfr_	48.7 ± 5.5
*F. akitsckensis*, budding stage	bud	FAEO_B_	46.5 ± 6.7
	leaf	FAEO_Lb_	47.8 ± 4.7
	root	FAEO_Rb_	N.A.
*F. akitsckensis*, fruiting stage	umbels with seeds	FAEO_u/s_	N.A.
	stem	FAEO_stm_	N.A.
	root	FAEO_Rfr_	N.A.
Major constituents	(±)-α-pinene		68.6 ± 7.9
	(1*S*)-(−)-β-pinene		51.4 ± 4.1
	(±)-sabinene		91.5 ± 13.6

N.A., no activity was observed, even at the highest tested concentration (100 μg/mL). IC_50_ values are presented as the mean ± S.D. of three independent experiments.

**Table 3:** Correlation coefficients of a linear regression analysis between antibacterial activity of the EOs and their compound composition based on GC-MS data.

Major Constituents/Chemical Class		Antibacterial Activity of EOs Expressed As	
		Log[IC_50_]	1/[IC_50_]
		Spearman Rank Correlation Coefficient (r) and Significance Level (p)	
Compound	α-pinene	−0.64 (*p* < 0.01)	0.26 (n.s. ^a^)
	β-pinene	−0.37 (n.s.)	−0.27 (n.s.)
	α/β-pinenes	−0.62 (*p* < 0.01)	0.21 (n.s.)
	sabinene	−0.29 (n.s.)	0.13 (n.s.)
	β-phellandrene	−0.13 (n.s.)	0.03 (n.s.)
	β-caryophyllene	−0.15 (n.s.)	0.51 (*p* < 0.03)
	*trans*-verbenol	−0.76 (*p* < 0.001)	0.74 (*p* < 0.002)
	eremophilene	−0.81 (*p* < 0.0001)	0.76 (*p* < 0.0001)
	2-himachalen-7-ol	−0.07 (n.s.)	0.07 (n.s.)
Chemical Class	monoterpene hydrocarbons	−0.50 (*p* < 0.05)	0.13 (n.s.)
	oxygenated monoterpenes	−0.08 (n.s.)	0.27 (n.s.)
	sesquiterpene hydrocarbons	−0.85 (*p* < 0.0001)	0.76 (*p* < 0.0001)
	oxygenated sesquiterpenes	−0.47 (n.s.)	0.32 (n.s.)

Concentration of compound(s) in EO samples are expressed as relative %. ^a^ n.s., no correlation (*p* > 0.05).
